# Sphaerocyclamide, a prenylated cyanobactin from the cyanobacterium *Sphaerospermopsis* sp. LEGE 00249

**DOI:** 10.1038/s41598-018-32618-5

**Published:** 2018-09-28

**Authors:** Joana Martins, Niina Leikoski, Matti Wahlsten, Joana Azevedo, Jorge Antunes, Jouni Jokela, Kaarina Sivonen, Vitor Vasconcelos, David P. Fewer, Pedro N. Leão

**Affiliations:** 1 Interdisciplinary Center of Marine and Environmental Research (CIIMAR/CIMAR), University of Porto, Terminal de Cruzeiros do Porto de Leixões, Avenida General Norton de Matos, S/N, 4450-208 Matosinhos, Portugal; 2 Faculty of Sciences, University of Porto, Rua do Campo Alegre, Porto, 4169-007 Portugal; 3 Department of Microbiology, PO Box 56, Viikki Biocenter, Viikinkaari 9, FI-00014, University of Helsinki, Helsinki, Finland

## Abstract

Cyanobactins are a family of linear and cyclic peptides produced through the post-translational modification of short precursor peptides. A mass spectrometry-based screening of potential cyanobactin producers led to the discovery of a new prenylated member of this family of compounds, sphaerocyclamide (1), from *Sphaerospermopsis* sp. LEGE 00249. The sphaerocyclamide biosynthetic gene cluster (*sph*) encoding the novel macrocyclic prenylated cyanobactin, was sequenced. Heterologous expression of the *sph* gene cluster in *Escherichia coli* confirmed the connection between genomic and mass spectrometric data. Unambiguous establishment of the orientation and site of prenylation required the full structural elucidation of 1 using Nuclear Magnetic Resonance (NMR), which demonstrated that a forward prenylation occurred on the tyrosine residue. Compound 1 was tested in pharmacologically or ecologically relevant biological assays and revealed moderate antimicrobial activity towards the fouling bacterium *Halomonas aquamarina* CECT 5000.

## Introduction

Cyanobacteria, in particular those belonging to the orders Oscillatoriales and Nostocales, are a rich source of bioactive secondary metabolites, many of which are polyketides or non-ribosomal peptides produced on modular enzymatic complexes^1,2^. Cyanobacteria also synthesize a range of natural products through the post-translational modification of short precursor peptides^2–7^. These are termed ribosomally synthesized and post-translational modified peptides (RiPPs)^3–5^. Biosynthetic gene clusters encoding RiPPs are broadly distributed among the cyanobacteria phylum^6^. Cyanobactins are a group of cyclic and linear RiPPs of cyanobacterial origin that have received attention due to their reported bioactivities and the biotechnological potential of their biosynthetic pathways^7,8^. Many cyanobactins were discovered as the result of anticancer activity screening programs and have general cytotoxicity against distinct tumor cell lines^8^. In addition, multidrug reversing (MDR), antiviral, antimalarial and allelopathic activities have been reported for these compounds^7–9^. Cyanobactin biosynthetic gene clusters encode precursor peptides that contain the core peptide sequence and act a as a substrate for post-translational modifications^8^. These commonly include heterocyclization of Cys, Ser and Thr residues, and oxidation of the resulting heterocycles to oxazoles and thiazoles^8,10^. Some cyanobactin biosynthetic pathways do not feature heterocyclases or oxidases and the resulting peptides do not contain such modified residues^8^. Isoprenoid derivatizations of Ser, Thr, Tyr or Trp residues, and more rarely *N*- or *O*-methylation^8,10,11^ are also sometimes reported for cyanobactins^8,10,11^. Prenylation can be forward, as observed for example in the prenylagaramides^12^, or reversed, as found for example in some aestuaramides^13^.

The core peptide of most cyanobactins is macrocyclized before release, although a number of highly modified linear cyanobactins have been reported^11^. The macrocyclic nature of cyanobactins makes them appealing for biotechnological use. Cyclic peptides have a higher permeability across membrane barriers, greater resistance to enzymatic degradation, enhanced bioavailability and higher affinity in receptor binding when compared to linear peptides^14,15^. However, studies regarding the bioactive potential and/or ecological role of a large fraction of cyanobactins are lacking. The main reason is likely related to the low amounts of isolated compounds, which are not enough to fully explore their biological activity potential.

**Figure 1:**
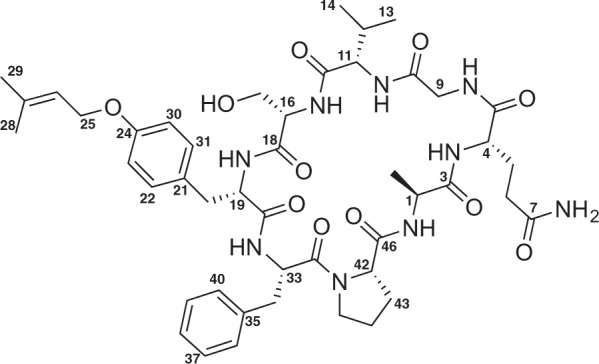
Structure of sphaerocyclamide (**1**).

Previous efforts to detect potentially novel cyanobactin biosynthesis genes in cyanobacterial strains from the Blue Biotechnology and Ecotoxicology Culture Collection (LEGE CC) revealed a number of strains likely to produce new cyanobactins^16^. Here we report the detection, isolation, structural elucidation and biological activity of sphaerocyclamide, a novel cyanobactin (**1**, Fig. 1).

## Results and Discussion

### LC-MS-based detection of a prenylated cyanobactin

An LC-MS^2^-based search for the presence of prenylated cyanobactins was performed on crude extracts of cyanobacterial strains known to contain PatA homologues, presumably involved in the maturation of cyanobactins^16^. A prenylated cyanobactin candidate was detected (*m/z* 918 for [M + H]^+^, abundant prenyl neutral loss (*m/z* 68) in MS^2^ spectrum, see Supplementary Information S1) in a methanol extract of the cyanobacterium *Sphaerospermopsis* sp. LEGE 00249, prompting us to further investigate this strain.

### Identification of the *sph* gene cluster in *Sphaerospermopsis* sp. LEGE 00249

**Figure 2:**
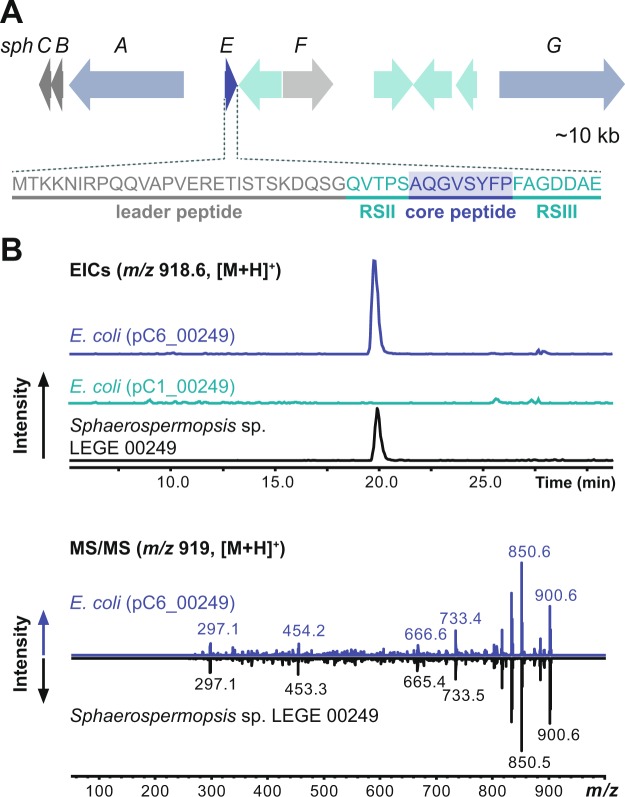
Biosynthesis of **1** in *Sphaerospermopsis* sp. LEGE 00249. (**A**) The sphaerocyclamide (*sph*) biosynthetic gene cluster is organized in a bidirectional operon. Genes marked in green encode conserved and hypothetical proteins that have no recognized or predicted function; the SphE precursor peptide is marked in dark blue. The genes encoding cyanobactin proteases are marked in light blue. The prenyltransferase gene is marked in light grey and genes commonly found in cyanobactin gene clusters but without predicted function are shown in dark grey. A transposase is present between *sphE* and *sphF* genes. Cyanobactin precursor peptides encode hypervariable amino acid core sequences. The precursor peptide core that originates **1** is shown below the *sphE* gene. (**B**) Comparison of the extracted ion chromatograms ([M + H]^+^, **1**) of methanol extracts of *Sphaerospermopsis* sp. LEGE 00249 (bottom), *E. coli* (pC6_00249) (top) and *E. coli* (pC1_00249) (middle, negative control) and comparison of MS^2^ data from *m/z* 919 ions (corresponding to **1**, [M + H]^+^) obtained from the *Sphaerospermopsis* sp. LEGE 00249 extract (bottom) and from *E. coli* (pC6_00249) (top).

An anacyclamide-like gene cluster was amplified from *Sphaerospermopsis* sp. LEGE 00249 by PCR, cloned and sequenced. The resulting *sph* gene cluster was found to encode 10 proteins (Table 1), including the SphA protease, SphG macrocyclase; SphE precursor peptide, SphF prenyltransferase, as well as the SphB and SphC proteins. Genes encoding a heterocyclase or oxidase were not detected. The SphA and SphG proteins are homologous to the proteases and macrocyclases involved in the biosynthesis of others cyanobactins. The SphF protein is homologous to other prenyltransferases present in cyanobactin pathways^10,17^. Likewise, SphB and SphC proteins are homologous to proteins encoded in almost all the cyanobactin gene clusters but with an unknown function^11^. The SphE precursor peptide, composed of 47 amino acids is highly homologous to a *Nodularia spumigena* precursor peptide and presents a new core sequence of eight amino acids (AQGVSYFP), flanked by recognition sequences RSII and RSIII^18^ (Fig. 2A). This information, together with the LC-MS data, enabled us to propose that the encoded cyanobactin is composed of a cyclic AQGVSYFP core structure decorated with a single prenyl moiety.

**Table 1:** Annotation of the *sph* gene cluster from *Sphaerospermopsis* sp. LEGE 00249.

*Sphaerospermopsis* sp. LEGE 00249		Blastp results^a^			
Protein	Predicted function	Description	Identity/similarity (aa %)	Organism	Accession number
SphC	Unknown	Cyanobactin biosynthesis system PatC/TenC/TruC family protein	94/94	*Nodularia spumigena* (several strains)	WP_006198022
SphB	Unknown	Cyanobactin biosynthesis system PatB/AcyB/McaB family protein	97/98	*Nodularia spumigena* (several strains)	WP_0066198021
SphA	N-terminal protease	PatA/PatG family cyanobactin maturation protease	93/95	*Nodularia spumigena* CENA596	WP_063871509
SphE	Precursor peptide	Anacyclamide/piricyclamide family prenylated cyclic peptide	81/95	*Nodularia spumigena* CENA596	WP_063871506
ORF5	Putative transposase	Transposase	92/93	*Aphanizomenon flos-aquae* 2012/KM1/D3	WP_039204680
SphF	Prenyltransferase	LynF/TruF/PatF family peptide O-prenyltransferase	98/98	*Dolichospermum circinale* AWQC131C	WP_028090374
ORF6	Putative endonuclease	Uma2 family endonuclease	97/98	*Cuspidothrix issatschenkoi* CHARLIE-1	WP_104387878
ORF7	Hypothetical protein	Hypothetical protein	98/98	*Dolichospermum circinale* AWQC131C	WP_028090379
ORF8	XisI protein	XisI protein	99/99	*Nodularia spumigena* CCY9414	WP_006196086
SphG	C-terminal protease	PatA/PatG family cyanobactin maturation protease	95/97	*Anabaena* sp. 90	WP_015081385

^a^Protein-protein Blast against the Refseq database, accessed May 2018.

### Heterologous expression of the *sph* gene cluster

To confirm the involvement of the *sph* gene cluster in the production of compound **1**, the entire *sph* gene cluster was expressed in *Escherichia coli* TOP10. Compound **1** (*m/z* 918) was detected by LC-MS in the cell extracts of *E. coli* TOP10 carrying plasmid pC6_00249 (which contained the *sph* genes), as confirmed by comparison of LC-MS and MS^2^ data with that of **1** found in *Sphaerospermopsis* sp. LEGE 00249 (Fig. 2B). The successful heterologous expression of **1** in *E. coli* confirmed that the *sph* gene cluster that we identified is responsible for the biosynthesis of this molecule.

### MS-guided isolation of 1

Bioinformatics analysis coupled with MS data indicated the core amino acid sequence and the presence of a prenyl group. However, the precise location and orientation of the prenyl unit was not directly inferable from the afore-mentioned data. Two amino acids in the core sequence could potentially be prenylated (Tyr and Ser)^5^ while *N*-prenylation^11^ at the Gln residue, although unprecedented in cyanobactins, could not be entirely discarded. Furthermore, normal and reverse-prenylations have been documented for this class of compounds^19^. Our first approach to clarify the position of the prenyl moiety was to analyze the MS/MS fragmentation patterns of **1**. Unfortunately, this analysis was unsuccessful (data not shown) owing to the adjacent positioning of Tyr and Ser in the structure of **1** and no fragmentations could unambiguously identify the precise location of the prenyl group. Therefore, to fully elucidate the structure of **1**, we obtained 1D and 2D NMR data that required prior purification of the compound. To achieve this, a methanol crude extract of *Sphaerospermopsis* sp. LEGE 00249 lyophilized cells (14.4 g, d.w.) was fractionated in a RP-SPE cartridge. The [M + H]^+^ ions corresponding to **1**, from LC-MS analysis, were mostly present in a single fraction that was further separated using analytical-scale RP-HPLC, to yield pure **1** (1.2 mg, 0.008% d.w.) (Supplementary Information S2).

### Structural elucidation of sphaerocyclamide using NMR reveals the prenylated residue

The ^1^H-NMR spectrum of **1** (Supplementary Information S3 and S4) was characteristic for a peptide, with discrete broad signals clearly corresponding to exchangeable (NH) protons (δ_H_ 8.81-6.72), α-methine multiplets (δ_H_ ~5.0-3.0) as well as the signature for the *gem*-dimethyl moiety of the prenyl group with two sharp singlets, each corresponding to three protons, resonating at δ_H_ 1.73 and 1.69, and revealing a forward prenylation in **1**. Due to the low amount of material and the size of the compound, we were unable to obtain high-quality ^13^C-data for **1**, although many characteristic signals were clearly discernible (Supplementary Information S3 and S5), including those corresponding to amide (δ_C_ 174.5-168.2), aromatic or olefinic (δ_C_ 157.2-114.4), as well as α-carbon (δ_C_ ~ 62.5-50.0) resonances.

**Figure 3:**
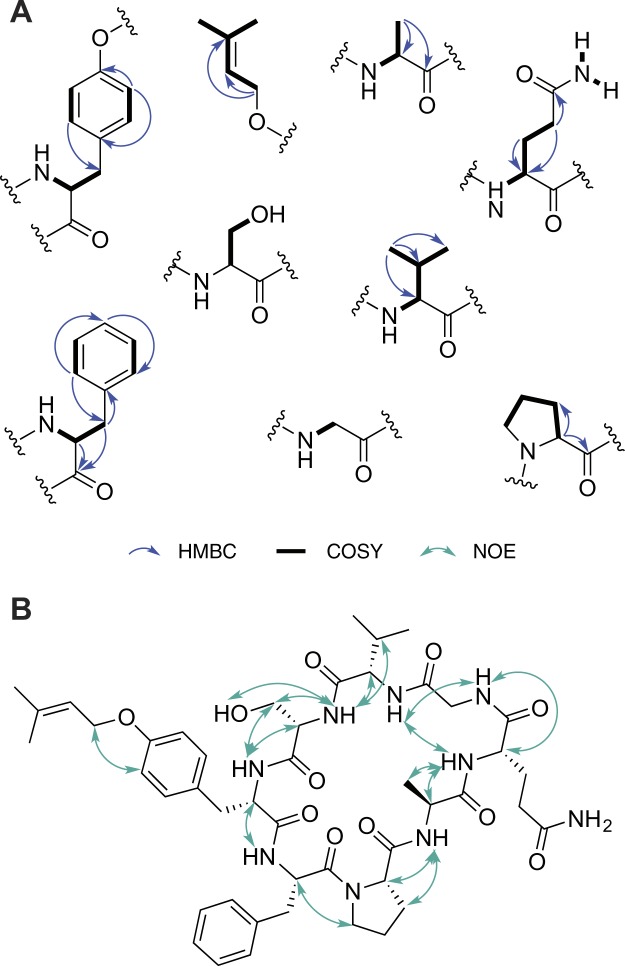
NMR-based structure analysis of **1**. (**A**) Selected HMBC and COSY correlations establishing the structures of the amino acids and prenyl group of **1**; (**B**) NOE-derived inter-residue connectivity and attachment of the prenyl group to the Tyr moiety.

Extensive 2D NMR analysis, including HSQC, HMBC and COSY (Supplementary Information S3, S6–S8), was carried out and used to assign spin systems for all the eight amino acid moieties and for the prenyl group that constitute compound **1** (Fig. 3A). ^1^H and ^13^C resonances were in agreement with the values from the literature for other cyclic peptides^12,20^. NOE data also allowed the connectivity of the eight amino acids residues to be determined (Supplementary Information S3 and S9), validating the bioinformatics predictions from analysis of the *sph* biosynthetic gene cluster (Fig. 3B). The NMR analysis provided strong evidence for the prenylation of Tyr, chiefly by exclusion of other possible sites for this modification. COSY data correlated the Ser H_2_17 proton (δ_H_ 3.54) with an exchangeable proton (OH-17, δ_H_ 5.11), excluding prenylation at this position. Two exchangeable, COSY-correlated proton signals (NH_a_-7 and NHb-7, δ_H_ 7.05 and 6.61, respectively) were highly diagnostic for the Gln side chain amide protons. In addition, because all the peptide backbone NH protons could be assigned from COSY, with the exception of the Ser NH which was assigned on the basis of extensive NOE correlations to Ser and Val protons, the possibility of *N*-prenylation was dismissed. Finally, aromatic protons H23 and H30 (δ_H_ 6.83) from the Tyr residue were found to correlate strongly through space with the H_2_25 (δ_H_ 4.47) protons from the prenyl moiety (Fig. 3B), establishing the planar structure of **1**. Taking into account the absence of an epimerase or racemization domain within the gene cluster encoding **1**, an l-configuration for all chiral amino acid residues was proposed and subsequently confirmed by Marfey’s analysis.

Compound **1** is structurally related to the anacyclamides, kawaguchipeptins, piricyclamides and prenylagaramides in that it is prenylated and lacks heterocyclized residues. Phylogenetic analysis based on the cyanobactin protease (Supplementary Information S10) demonstrates that the biosynthetic gene cluster of metabolite **1** is significantly closer to the anacyclamides than to the prenylagaramides. Known anacyclamides have between 7 and 20 proteinogenic amino acids in their cyclic structure^21^. Anacyclamides are produced in low amounts and it remains unclear which positions on these anacyclamide cores are prenylated^19^. In the prenylagaramides, as in compound **1**, the Tyr residues were found to be prenylated, so it is possible that prenylated anacyclamides containing Tyr share this modified residue^12^. Nervertheless, given the multiple isoprenoid decorations that are found in cyanobactins, it is still not feasible to accurately predict, exclusively from genetic information, the type and site of prenylation in these peptides.

### Biological activity of 1

Several bioactivities have been attributed to cyanobactins^7–9^. However, there is no biological activity reported in the literature for any of the anacyclamides or prenylagaramides. Cyanobacterial cyclic peptides containing only unmodified proteinogenic amino acids present some relevant pharmaceutical activities. *Planktothrix* spp. produce agardhipeptin A, a weak plasmin inhibitor^22^, and planktocyclin, a potent protease inhibitor of mammalian trypsin and α-chymotrypsin^23^. This cyanobacterium is also responsible for the production of the prenylated prenylagaramides, but no activity has been assigned to these cyclic peptides^12,24^. *Microcystis aeruginosa* strains produce unmodified and prenylated forms of the cyanobactin kawaguchipeptin, with reported antibacterial activity^10,25,26^, and piricyclamides^27^, although no bioactivity has been determined for the latter. We selected several bioassays to test the potential bioactivity of sphaerocyclamide. No relevant cytotoxic, anti-inflammatory, anti-obesity, anti-quorum sensing or allelopathic activities as well as HDAC or 20S proteasome inhibition were found. Antibacterial activity towards clinically-relevant isolates was also not observed. However, compound **1** weakly inhibited the growth of *Halomonas aquamarina* (CECT 5000), a biofilm-forming marine biofouling bacterium. The MIC for this interaction was estimated to be 240 μg mL^−1^, a value above the highest tested concentration (128 μg mL^−1^, Supplementary Information S11). The corresponding EC_50_ value was 23.1 μg mL^−1^ (25.2 μM, Supplementary Information S11). Albeit mild, this antibacterial activity is in line with other antifoulant natural products such as the diterpene dictyol C^28^ or the bromopyrrole oroidin^29^. Compound **1** was more effective in inhibiting *H. aquamarina* than a series of natural scaffold-inspired antifouling sulfated compounds^30^. Due to the complex nature of biofouling phenomena^31,32^, future work will be carried out to evaluate whether **1** can act as an antifoulant, especially in light of the low toxicity this compounds exhibits towards mammalian cells.

To conclude, we herein describe the discovery of sphaerocyclamide (**1**), in the cyanobacterium strain *Sphaerospermopsis* sp. LEGE 00249. To our knowledge this is the first report of a cyanobactin isolated from this recently established genus, reassigned together with the genera *Dolichospermum*^33^ (that includes some of the anacyclamide-producing strains reported to date) and *Cuspidothrix* from the genus *Anabaena*. Despite their apparently elusive biological activities, the structural group cromprising sphaerocyclamide, piricyclamides, kawaguchipeptins, anacyclamides and prenylagaramides, and the cyanobactins in general are highly diverse and we appear to have isolated only a small fraction of their abundance^6,11^. Efforts to purify and characterize novel members of these families are therefore warranted and may reveal novel chemistry, enzymology and bioactivity.

## Methods

### Cyanobacterial strain culture conditions

The freshwater strain *Sphaerospermopsis* sp. LEGE 00249 was isolated from Maranhão reservoir, Portugal (N39°03′16,6″, W7°54′42,0″)^16^. A culture of this strain is maintained in the LEGE CC in Z8 medium^34^ without aeration, at 20 °C, under a light/dark cycle of 14:10 h and a photon irradiance of approximately 10 μmol m^−2^ s^−1^. *Sphaerospermopsis* sp. LEGE 00249 cells were grown for approximately one month to late exponential phase with aeration at 25 °C in multiple 6 L glass flasks (containing 4 L of Z8 medium), under a light/dark cycle of 14:10 h and a photon irradiance of approximately 30 μmol m^−2^ s^−1^ for biomass extraction and subsequent isolation of **1**. The cultures were harvested by centrifugation (Sorvall Legend RT, 4500 × *g*, 10 min) after reaching the stationary phase, rinsed with deionized water and lyophilized.

### LC-MS-based detection of prenylated cyanobactins

Lyophilized biomass (~10 mg) from each of several LEGE CC cyanobacterial cultures, for which the presence of the cyanobactin biosynthesis *A* gene had been previously reported^16^, was extracted with 1 mL of MeOH in 2-mL microcentrifuge tubes containing glass beads (cell disruption medium, 0.5-mm-diameter glass beads, Scientific Industries Inc.) and homogenized with a FastPrep cell disrupter (Bio101, Thermo Electron Corporation, Qbiogen, Inc.) for 15 s at a speed of 6.5 m s^−1^. Each sample was centrifuged for 5 min at 20,000 × *g* (Eppendorf Centrifuge 5840R, Eppendorf), and the supernatant analyzed using a high-performance liquid chromatograph combined with a mass spectrometer (LC-MS) (Agilent 1100 series LC/MSD with Ion Trap XCT Plus and an electrospray ion source) in order to detect low-molecular-weight peptides. Separation of peptides was performed by HPLC equipped with a Luna C_8_(2) column (Phenomenex; 2.0 mm by 150 mm; particle size 5 μm). The mobile phase consisted of 0.1% aqueous formic acid (50% solution in water; Fluka, Sigma-Aldrich) (solvent A) and 0.1% formic acid in isopropyl alcohol (Sigma-Aldrich) (solvent B). A gradient program from 5% to 100% of solvent B during 35 minutes was used at a flow rate of 0.15 mL min^−1^. The column was heated during separation to 40 °C and the positive-ion mode of electrospray ionization was used. The nebulizer gas (N_2_) pressure was 30 lb/in^2^, the flow rate of the drying gas was 8 L min^−1^ and the temperature was 350 °C. The capillary voltage was set at 5,000 V, end plate offset −500 V and the capillary exit value was 300 V. A skimmer potential of 85 V and a trap drive value of 144 were used. Spectra were recorded using a scan range from *m/z* 100 to *m/z* 2,200.

### Identification of the *sph* gene cluster in *Sphaerospermopsis* sp. LEGE 00249

Biomass from 40 mL culture of *Sphaerospermopsis* sp. LEGE 00249 grown until exponential-phase was harvested by centrifugation at 7,000 × *g* for 7 min (Eppendorf Centrifuge 5840R, Eppendorf). Total genomic DNA was extracted from the resulting pellet using a commercial kit (E.Z.N.A plant DNA mini kit, Omega Bio-Tek, Doraville, GA) and following the manufacturer’s procedures. The *sphE* peptide precursor gene was amplified using primers preRNAF (5′GAAG AACATCCGCCCCCAACAAGTTG3′) and preRNAR (5′CTCCGCGTCGTC GCCTGCAAAAGG3′) and primers PreF (5′GCCTTCACCAAACCAGTCTTCTTCAT3′) and PreR (5′CATCGAGGCGAACCGTGCGCCAAGGGAT3′). PCR amplifications were performed as previously described^21^. Sequencing data was obtained using the BigDye® Terminator Cycle Sequencing Ready Reaction kit (version 3.1) (PE Applied Biosystems) and analyzed on an ABI 3730xl automated sequencer.

The entire *sph* gene cluster was amplified from the genomic DNA of *Sphaerospermopsis* sp. LEGE 00249 by PCR using primers acyCF (5′ATTAACACCAAATACGACCACGACGG3′) and acyGR (5′TTATGCTGATTTTGCCACAGACCAAGAACG3′) in six 20 μL reaction mixtures containing 5× Phusion GC Buffer (BioLabs Inc.), 200 μmol of each deoxynucleotide triphosphate (Thermo Fisher Scientific), 0.75 μM of each of the primers, 0.8 U of Phusion High-Fidelity DNA Polymerase (BioLabs Inc.), and 61.9 ng of *Sphaerospermospsis* sp. LEGE 00249 template DNA. The thermal cycling conditions were as follows: 94 °C for 3 min, followed by 30 cycles of 94 °C for 30 s, 53.1 °C for 30 s, and 68 °C for 11 min and then a final extension at 68 °C for 20 min. The PCR products were separated in a 0.6% agarose gel in 0.5× TAE buffer stained with SYBR Safe DNA gel stain (Invitrogen) and visualized using a Dark reader (Clare Chemical Research Inc.) to avoid DNA cross-linking from UV light during gel excision. The 10-kb PCR product was gel extracted using the MinElute gel extraction kit (Qiagen) and cloned into the pCR^TM^2.1-TOPO vector using a TOPO TA cloning kit (Invitrogen) with an insert-to-vector ratio of 3:1. Chemically competent *Escherichia coli* One Shot TOP 10 cells (Invitrogen) were transformed with the vector according to the manufacturer’s instructions. The resultant plasmids (pC4_00249, pC6_00249, pC7_00249, pC8_00249 and pC16_00249) were analyzed by PCR and restriction enzyme digestions were carried out to ensure the maintenance and integrity of the insert during cloning and amplification in *E. coli*. The transformants containing the *sph* gene cluster insert in the plasmid were grown overnight with shaking at 120 rpm at 37 °C in 50 mL of LB medium containing 100 μg mL ^−1^ of ampicillin (sodium salt; Sigma-Aldrich) for LC-MS analysis. The cells (33.4–41.4 mg) were harvested by centrifugation at 7,000 × *g* for 7 min (Eppendorf Centrifuge 5840 R, Eppendorf), extracted with MeOH and the resulting extracts were directly analyzed by LC-MS as detailed for cyanobacterial cell extracts. Plasmid pC6_00249 was sequenced and the *sph* gene cluster was obtained after LC-MS analyses of *E. coli* transformed cells. The gene cluster was manually annotated in Artemis (Sanger Institute) using a combination of BLASTp and CD (Conserved Domains) database searches. Glimmer was used for open reading frame (ORF) predictions and BLASTp was performed to evaluate the functional analysis of the proteins. The predictions for start sites of the genes were checked manually. The annotated sequence of the *sph* gene cluster from *Sphaerospermopsis* sp. LEGE 00249 was deposited in GenBank under the accession number MF140698.

### Extraction and LC-MS-Guided Isolation of 1

Lyophilized biomass (14.4 g, d.w.) of the cyanobacterium *Sphaerospermopsis* sp. LEGE 00249 was repeatedly extracted with MeOH yielding 2.6 g of crude extract. A portion of the crude extract (0.80 g) was fractionated using a C18-E SPE cartridge (50 g, Strata, Phenomenex) with a stepwise gradient from 10% MeCN (aq) to 100% MeCN to 100% acetone, yielding eighteen fractions. These were subjected to Electrospray Ionization (ESI) LC-ESI-MS profiling (Alliance e2695 separations module coupled to a Micromass Quattro Micro Api™ detector) in order to search for the predicted mass of **1** (*m/z* 918) [M + H]^+^. Separation was performed by HPLC equipped with an Aeris Peptide C_18_ column (Phenomenex; 4.6 mm by 150 mm; particle size, 3.6 μm). The mobile phase consisted of MeCN (solvent A) and MilliQ water (solvent B) both acidified with 0.1% formic acid. A linear gradient program from 5% to 70% of solvent A during 20 minutes, and then returning to the initial conditions in a total run time for 30 min, was used at a flow rate of 0.15 mL min^−1^. The column was heated during separation to 25 °C and the positive-ion mode of electrospray ionization was used. The nebulizer gas (N_2_) flow rate was 500 liters hr^−1^ and the desolvation temperature was 250 °C. Spectra were recorded using single ion recording (SIR) of *m/z* 918 in a scan range from *m/z* 30 to 1000. Collected data was interpreted using MassLynnx™ software (Waters).

One of the fractions (13.1 mg), eluting with 50% MeCN (aq), presented the highest amount of **1** (Supplementary Information S2) and was therefore further separated by HPLC (using a system composed of a 1525 pump coupled to a 2487 UV-Vis detector, Waters) fitted with a Synergy 4 μ Fusion RP-80A column (Phenomenex; 4.6 mm by 250 mm; particle size 4 μm). A gradient program from 5% MeCN (aq) to 100% MeCN during 35 minutes, then held for 5 minutes before returning to the initial conditions over 5 minutes, was used with a flow rate of 1 mL min^−1^ and the separation was monitored at two different wavelengths (235 and 280 nm). The resulting fractions were submitted to LC-ESI-MS analysis, in order to search for the presence of **1**. Analysis conditions were as previously described for the analysis of the SPE fractions, with the exception of the gradient program, that consisted in an increase from 40% to 80% of solvent A during 10 minutes, and then returning to the initial conditions in a total run time for 15 minutes, at flow rate of 0.3 ml min^−1^. One of the HPLC subfractions (t_R_ = 19. 5 min) presented the correspondent predicted mass (Supplementary Information S2). Therefore, successive rounds of HPLC separation as described above were performed until **1** was obtained (1.2 mg, 0.03% d.w.) with enough purity for structural analysis and bioactivity testing.

Sphaerocyclamide (**1**): white amorphous solid; UV (MeOH) λmax (log ε) 206 (3.8), 226 (3.6), 268 (3.0), 278 (3.0) nm; ^1^H, ^13^C NMR, HMBC, COSY and NOESY data see Supplementary Information S3–S9; HRESIMS (see Supplementary Information S12) *m/z* 940.4545 [M + Na]^+^ (calcd for C_46_H_63_N_9_O_11_Na^+^, 940.4539).

UV-Vis data was acquired on a microplate reader (Synergy HT, Biotek, USA). Optical rotation data was measured (average of ten replicates) on a Jasco P-2000 polarimeter at a wavelength of 589 nm using a 1 mL cell. NMR data were acquired in a 600 MHz Bruker Avance III equipped with a 5 mm cryoprobe. NMR spectra were referenced to the residual solvent ^1^H and ^13^C signals (δ_H_ 2.500 and δ_C_ 39.52 for DMSO, respectively). High-resolution mass spectrometry was performed on a LTQ Orbitrap XL spectrometer, controlled by LTQ Tune Plus 2.5.5. and Xcalibur 2.1 (Thermo Scientific). The capillary voltage of the ESI was set to 2800 V. The capillary temperature was 300 °C. The sheath gas and auxiliary gas flow rate (nitrogen) were set to 40 and 10 (arbitrary unit as provided by the software settings). The capillary voltage was −48 V and the tube lens voltage −247.79 V. The sample was injected at a concentration of approximately 50 μg mL^−1^. All solvents used were LC-MS-grade or HPLC-gradient grade for LC-MS-based experiments, HPLC gradient for HPLC analysis/purification and ACS grade for extraction and SPE, unless stated otherwise. NMR solvents were acquired either from Sigma-Aldrich or BDH Prolabo (VWR).

### Absolute configuration of 1

A hydrolysate of compound **1** (0.4 mg, 6 N HCl, 110 °C, 24 h) was dried under a N_2_ stream and dissolved in 50 μL of water before 20 μL of 1 M NaHCO_3_ was added. One-hundred microliters of a 1-fluoro-2,4-dinitrophenyl-5-l-alanine amide (FDAA, Marfey’s Reagent) solution in acetone (1%, w/v) was added and the mixture was incubated for 1 h at 37 °C. The reaction was stopped with 20 μL 1 M HCl. The analysis of FDAA amino acid derivatives was carried out with LCMS (Waters Acquity UPLC and Synapt G2-Si QTOF). A Kinetex® 1.7 µm C8 100 Å, LC Column 50 × 2.1 mm was used with a gradient of solutions A (0.1% HCOOH in water) and B (0.1% HCOOH in 2-propanol/acetonitrile 1:1). B was changed from 5% to 60% in 6.00 min then the column was washed with 100% of B for 2.00 min and equilibrated in 5% of B for 2.00 min. The retention times observed for the [M + Na]^+^ adducts of the FDAA amino acid derivatives of hydrolysate and standards (in brackets) were as follows: 2.80 min (d-Ala 3.12 min, l-Ala 2.80 min); 2.61 min (d-Glu 2.81 min, l-Glu 2.62 min); 2.45 min (Gly 2.44 min); 3.65 min (d-Val 4.09 min, l-Val 3.65 min); 2.17 min (d-Ser 2.21 min, l-Ser 2.17 min); 3.20 min (d-Tyr 3.40 min, l-Tyr 3.21 min); 4.01 min (d-Phe 4.36 min, l-Phe 4.01 min); 2.87 min (d-Pro 2.97 min, l-Pro 2.88 min).

### Biological activity evaluation of 1

#### Preparation of stock solutions

Stock solutions of **1** were prepared in DMSO (cell culture grade, Sigma-Aldrich) at 1 and 3 mg mL^−1^ and used in several bioassays. For MIC determination, DMSO stock solutions of **1** were prepared at different concentrations (0.0125–12.8 mg mL^−1^) by serial dilution.

#### Bioassays with fouling marine bacteria

Compound **1** was tested at a concentration of 10 μg mL^−1^ (11 μM) against five marine fouling bacteria strains, acquired from the Spanish Type Culture Collection (CECT). The bacteria *Cobetia marina* CECT 4278, *Vibrio harveyi* CECT 525, *Roseobacter litoralis* CECT 5395, *Halomonas aquamarina* CECT 5000, and *Pseudoalteromonas atlantica* CECT 570 were inoculated with **1** in Marine Broth (MB) (Difco) at an optical density (OD) 600 nm of ~0.1, in 96 well flat-bottom microtiter plates (Orange Scientific, Belgium), and grown at 26 °C for 24 hours. Bacterial growth in the presence of **1** was compared to the growth in MB medium by reading OD at 600 nm using a microplate reader (Synergy HT, Biotek, USA). An antibiotic cocktail (penicillin 5000 units mL^−1^, streptomycin 5 mg mL^−1^ and neomycin 10 mg mL^−1^, Sigma-Aldrich) was used as positive control whereas DMSO (1%, v/v) was used as a solvent control. When growth inhibition caused by **1** was observed (i.e. for *Halomonas aquamarina* CECT 5000), dose-response curves were created and MIC values calculated (Gompertz equation model, bottom fit values corresponding to the positive control OD) using Prism 7 (GraphPad Software). For comparative purposes, IC_50_ values were also determined (from a four-parameter equation fit) using the same software.

*Cancer cell cytotoxicity assays, anti-inflammatory activity assays, anti-obesity activity assays, HDAC and 20S proteasome inhibition assays, anti-quorum sensing activity test, antibacterial activity assays using clinically-relevant strains and allelopathic activity assays*. Please see Supplementary Information (S13).

## Electronic supplementary material

Supplementary Information

## Data Availability

The datasets generated during and/or analysed during the current study are available from the corresponding author on reasonable request.
