# Supplementary material for: Sphaerocyclamide, a prenylated cyanobactin from the cyanobacterium Sphaerospermopsis sp. LEGE 00249
Source: Sci Rep. 2018 Sep 28;8:14537. doi: 10.1038/s41598-018-32618-5 (PMC6162287; doi:10.1038/s41598-018-32618-5)
Supplement: Supplementary file 1 — Supplementary Information [file 41598_2018_32618_MOESM1_ESM.docx]

**Supplementary Information**

**Sphaerocyclamide, a prenylated cyanobactin from the cyanobacterium *Sphaerospermopsis* sp. LEGE 00249**

Joana Martins^a,b^, Niina Leikoski^c^, Matti Wahlsten^c^, Joana Azevedo^a,b^, Jorge Antunes^a,b^, Jouni Jokela^c^, Kaarina Sivonen^c^, Vitor Vasconcelos^a,b^**_,_** David P. Fewer*^c^, Pedro N. Leão*^a^

^a^ Interdisciplinary Center of Marine and Environmental Research (CIIMAR/CIMAR), University of Porto, Terminal de Cruzeiros do Porto de Leixões, Avenida General Norton de Matos, S/N, 4450-208 Matosinhos, Portugal.

^b^ Faculty of Sciences, University of Porto, Rua do Campo Alegre, Porto 4169-007, Portugal.

^c^ Department of Microbiology, PO Box 56, Viikki Biocenter, Viikinkaari 9, FI-00014, University of Helsinki, Finland.

**Fig. S1.** Neutral loss of a prenyl group in **1**, as detected in the LC-MS^2^ analysis of the methanol extract of *Sphaerospermopsis* sp. LEGE 00249.


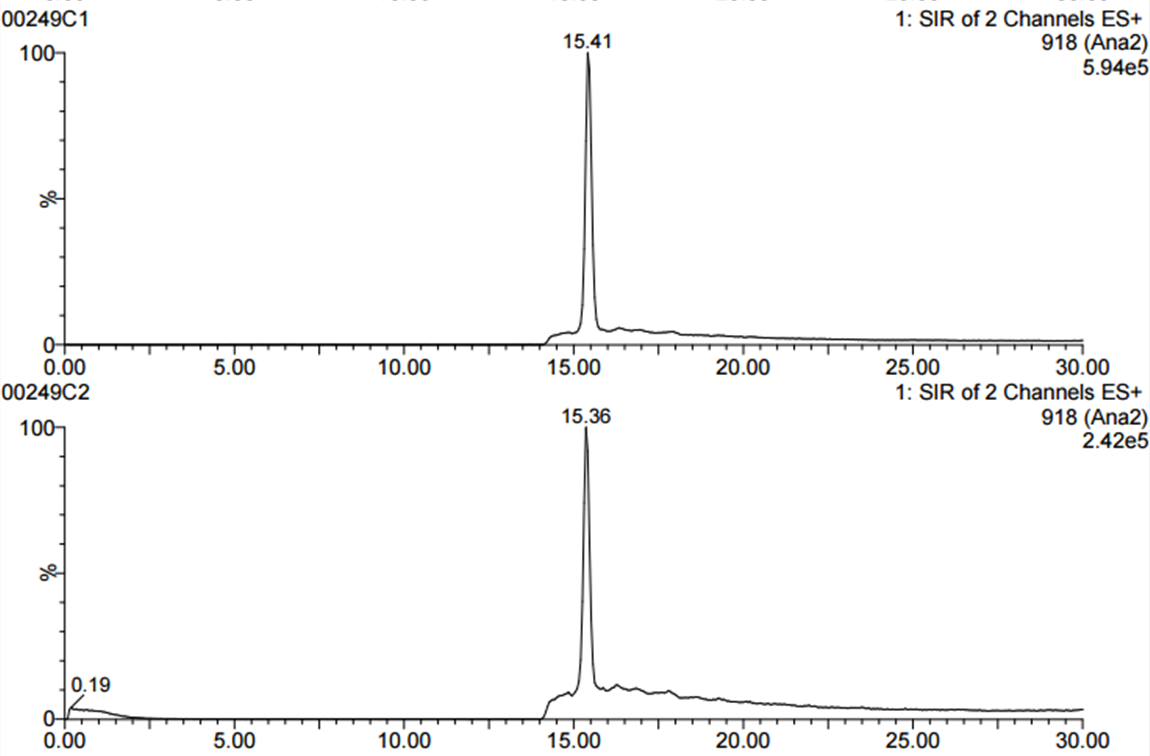


**Fig. S2.** Top - Selected Single Ion Recording (SIR) chromatogram (*m/z* 918) from SPE fractions from *Sphaerospermopsis* sp. LEGE 00249. SPE fraction 00249C1 was selected for further RP-HPLC purification of **1**; bottom - Selected Single Ion Recording (SIR) chromatograms (*m/z* 918) from HPLC fraction 00249C1_B from *Sphaerospermopsis* sp. LEGE 00249.

**Table S3.** NMR spectroscopic data (600 MHz for ^1^H and 150 MHz for ^13^C, DMSO-*d*_6_, 25 ºC) for sphaerocyclamide (**1**).

| **Unit** | **No** | **δ_C_** | **Group** |  | **δ_H_ [mult., *J* (Hz)]** | |  | **HMBC** | **COSY** | **NOESY** |
| --- | --- | --- | --- | --- | --- | --- | --- | --- | --- | --- |
| Ala | 1 | 51.3 | CH |  | 3.92 | m |  |  | 1NH, 2 | 1NH, 2, 4NH |
|  |  |  | NH |  | 8.57 | br s |  |  | 1 | 1 , 2, 4NH, 42, 43a, 43b, 45a, 45b |
|  | 2 | 16.3 | CH_3_ |  | 1.28 | d, 7.5 |  | 1, 3 | 1 | 1, 1NH, 4NH |
|  | 3 | 172.8 | C = O |  |  |  |  |  |  |  |
| Gln | 4 | 51.3 | CH |  | 4.26 | m |  |  | 4NH, 5a, 5b | 4NH, 5a, 5b, 6, 9NH, 11NH |
|  |  |  | NH |  | 7.78 | d, 9.6 |  |  | 4 | 1, 1NH, 2, 4, 5a, 6, 11NH |
|  | 5a | 26.5 | CH_2_ |  | 1.60 | m |  | 4 | 4, 5b, 6 | 4, 4NH, 5b, 6 |
|  | 5b |  |  |  | 2.29 | m |  | 7 | 4, 5a, 6 | 4, 5a, 6 |
|  | 6 | 31.2 | CH_2_ |  | 1.92 | t, 7.2 |  | 4, 5a, 7 | 5a, 5b | 4, 4NH, 5a, 5b |
|  | 7 | 173.0 | C = O |  |  |  |  |  |  |  |
|  | 7a |  | NH_2_ |  | 7.05 | br s |  |  | 7b | 7b |
|  | 7b |  |  |  | 6.61 | br s |  |  | 7a | 7a |
|  | 8*^a^* | 171.5 | C = O |  |  |  |  |  |  |  |
| Gly | 9a | 42.9 | CH_2_ |  | 3.36 | m |  |  | 9b, 9NH |  |
|  | 9b |  |  |  | 3.87 | dd, 6.3, 16.9 |  | 10*^a^* | 9a, 9NH | 9NH, 11NH |
|  |  |  | NH |  | 7.86 | t, 6.3 |  | 8*^a^* | 9a, 9b | 1NH, 4, 9b, 11NH, 42 |
|  | 10*^a^* | 168.2 | C = O |  |  |  |  |  |  |  |
| Val | 11 | 54.5 | CH |  | 4.71 | m |  |  | 11NH, 12 | 11NH, 12, 13, 14, 16NH |
|  |  |  | NH |  | 6.72 | d, 10.4 |  |  | 11 | 4, 4NH, 9b, 9NH, 11CH, 12, 13, 14, 42 |
|  | 12 | 31.8 | CH |  | 2.03 | m |  |  | 11, 13, 14 | 11, 11NH, 13, 14, 16NH |
|  | 13 | 19.3 | CH_3_ |  | 0.76 | d, 6.8 |  | 11, 12, 14 | 12 | 11, 11NH, 12, 16NH |
|  | 14 | 16.4 | CH_3_ |  | 0.54 | d, 6.7 |  | 11, 12, 13 | 12 | 11, 11NH, 12, 19NH, 34b, 16NH |
|  | 15*^b^* | 173.8 | C = O |  |  |  |  |  |  |  |
| Ser | 16 | 59.1 | CH |  | 3.73 | m |  | 18*^b^* | 17 | 16NH, 19NH |
|  |  |  | NH |  | 8.81 | br s |  | 15*^b^* |  | 11, 12, 13, 16, 17CH2, 19NH, 14 |
|  | 17 | 60.1 | CH_2_ |  | 3.54 | m |  |  | 16, 17OH | 16NH, 19NH |
|  |  |  | OH |  | 5.11 | m |  |  | 17 |  |
|  | 18*^b^* | 170.3 | C = O |  |  |  |  |  |  |  |
| O-Prenyl Tyr | 19 | 54.9 | CH |  | 4.11 | m |  |  | 19NH, 20b | 19NH, 20a, 20b, 22, 31, 33NH |
|  |  |  | NH |  | 7.46 | br s |  |  | 19 | 14, 16, 16NH, 17CH2, 19, 20a, 20b, 22, 31 |
|  | 20a | 33.9 | CH_2_ |  | 2.79 | dd, 4.1, 14.2 |  | 22/31 | 20b | 19, 19NH, 20b, 22, 31 |
|  | 20b |  |  |  | 3.15 | m |  | 19, 21, 22/31 | 19, 20a | 20a, 19CH, 19NH, 22/31 |
|  | 21 | 129.4 | C |  |  |  |  |  |  |  |
|  | 22 | 129.8 | CH |  | 7.02 | d, 8.5 |  | 20a, 23/30, 24, 31 | 23 | 19, 19NH, 20a, 20b, 23/30 |
|  | 23 | 114.4 | CH |  | 6.83 | d, 10.3 |  | 21, 24, 30 | 22 | 22/31, 25 |
|  | 24 | 157.2 | C |  |  |  |  |  |  |  |
|  | 25 | 64.2 | CH_2_ |  | 4.47 | m |  | 26, 27 | 26, 29 | 23/30, 26, 28 |
|  | 26 | 120.0 | CH |  | 5.41 | m |  | 25, 28, 29 | 25, 28, 29, | 25, 29 |
|  | 27 | 136.9 | C |  |  |  |  |  |  |  |
|  | 28 | 18.0 | CH_3_ |  | 1.69 | s |  | 26, 27, 29 | 26 | 25 |
|  | 29 | 25.4 | CH_3_ |  | 1.73 | s |  | 26, 27, 28 | 25, 26 | 26 |
|  | 30 | 114.4 | CH |  | 6.83 | d, 10.3 |  | 21, 23, 24 | 31 | 25, 22/31 |
|  | 31 | 129.8 | CH |  | 7.02 | d, 8.5 |  | 20a, 22, 23/30, 24 | 30 | 19, 19NH, 20a, 20b, 23/30 |
|  | 32 | N.A. | C = O |  |  |  |  |  |  |  |
| Phe | 33 | 52.2 | CH |  | 4.61 | dd, 7.5, 14.0 |  | 41 | 33NH, 34a, 34b | 33NH, 34a, 34b, 45a, 45b |
|  |  |  | NH |  | 7.41 | br s |  |  | 33 | 14, 19, 33, 34a, 45b |
|  | 34a | 35.6 | CH_2_ |  | 2.63 | dd, 5.3, 14.1 |  | 33, 35, 36/40, 41 | 33, 34b | 33, 33NH, 34b |
|  | 34b |  |  |  | 3.05 | m |  | 33, 35, 36/40, 41 | 33, 34a | 14, 33CH, 34a |
|  | 35 | 138.2 | C |  |  |  |  |  |  |  |
|  | 36 | 129.0 | CH |  | 7.20 | d,7.3 |  | 34, 40, 38 | 37 |  |
|  | 37 | 128.1 | CH |  | 7.27 | dd,7.3, 7.7 |  | 35, 39 | 36 |  |
|  | 38 | 126.0 | CH |  | 7.18 | d, 7.7 |  | 34, 38, 36/40 |  |  |
| **Table S3** (continued) | | |  |  |  |  |  |  |  |  |
| **Unit** | **No** | **δ_C_** | **Group** |  | **δ_H_ [mult., *J* (Hz)]** | |  | **HMBC** | **COSY** | **NOESY** |
|  | 39 | 128.1 | CH |  | 7.27 | dd,7.3, 7.7 |  | 35, 37 | 40 |  |
|  | 40 | 129.0 | CH |  | 7.20 | d,7.3 |  | 34, 36, 38 | 39 |  |
|  | 41 | 169.9 | C = O |  |  |  |  |  |  |  |
| Pro | 42 | 62.5 | CH |  | 4.22 | t, 8.0 |  | 43, 46 | 43a, 43b | 1NH, 9NH, 11NH, 43a, 43b |
|  | 43a | 28.1 | CH_2_ |  | 1.77 | m |  |  | 42, 43b | 1NH, 42, 43b |
|  | 43b |  |  |  | 2.15 | m |  |  | 42, 43a, 44a | 1NH, 42, 43a, 44b |
|  | 44a | 24.2 | CH_2_ |  | 1.49 | m |  | 43 | 43b |  |
|  | 44b |  |  |  | 1.88 | m |  |  | 45b | 43b, 45b |
|  | 45a | 46.7 | CH_2_ |  | 3.37 | m |  |  | 45b | 1NH, 33 |
|  | 45b |  |  |  | 3.60 | m |  |  | 44b, 45a | 1NH, 33, 33NH, 44b |
|  | 46 | 174.5 | C = O |  |  |  |  |  |  |  |

*^a, b^*denotes pairs (positions with same letter) of amide carbons and respective HMBC correlations which could not be unambiguously assigned and therefore the opposite assignment cannot be excluded.

N.A.: not assigned

**Fig. S4.** ^1^H NMR spectrum (600 MHz, DMSO-*d_6_*) of sphaerocyclamide (**1**).

**Fig. S5.** ^13^C NMR spectrum (150 MHz, DMSO-*d_6_*) of sphaerocyclamide (**1**).

**Fig. S6.** HSQC spectrum (600 MHz, DMSO-*d_6_*) of sphaerocyclamide (**1**).

**Fig. S7.** HMBC spectrum (600 MHz, DMSO-*d_6_*) of sphaerocyclamide (**1**).

**Fig. S8.** COSY spectrum (600 MHz, DMSO-*d_6_*) of sphaerocyclamide (**1**).

**Fig. S9.** NOESY spectrum (600 MHz, DMSO-*d_6_*) of sphaerocyclamide (**1**).

**Fig. S10.** Phylogenetic analysis of cyanobactin proteases. The amino acidic sequences from cyanobactin protease genes in publicly available genomes were aligned with SphA in the Geneious package (Biomatters) using the MUSCLE algorithm. The alignment was then used for the construction of a maximum-likelihood phylogenetic tree using RAxML 8.2.11 from within the Geneious software, employing rapid bootstrapping and subsequent ML search. The GAMMA model was sued with an LG substitution matrix and a total of 500 bootstrap inferences were performed. The best scoring ML tree is shown in the figure. Clades corresponding to anacyclamides and prenylagaramides are shaded. The SphA protease is shown in red whereas the prenylagaramide biosynthesis protease (PagA) is shown in blue.

**Fig. S11.** Dose-response curve for the *H. aquamarina* CECT 5000 bioassay.

**Fig. S12.** HR-ESIMS spectrum for sphaerocyclamide (**1**).

**Methods S13.** Experimental details

**Cancer cell cytotoxicity assays**

Cytotoxic activity of **1** was tested against the osteosarcoma (MG-63), breast cancer (T47D) and colon cancer (HT-29) cell lines. Cells were cultivated in Dulbecco’s modified Eagle medium (DMEM Glutamax, Invitrogen, CA, USA) supplemented with 10% fetal bovine serum (FBS, Biochrom, Berlin, Germany), 1% penicillin/streptomycin (10,000 IU mL^-1^ and 10 mg mL^-1^, respectively; Biochrom) and 0.1% amphotericin (GE healthcare, Little Chafont, United Kingdom). Cells were incubated at 37 ºC in a humidified atmosphere with 5% of CO_2_.The analysis of cytotoxicity was determined by the reduction of the 3-(4,5-dimethylthiazole-2-yl)-2,5-diphenyltetrazolium bromide (MTT, Sigma-Aldrich, St. Louis, MO, USA). Cells were seeded into 96-well plates at a density of 3.3 x 10^4^ cells mL^-1^ and incubated for 24 hours to allow cell adhesion. Cells were exposed to 100 μL of fresh medium containing **1** at a final concentration of 5 and 10 μM during a period of 48 hours. MTT was added with a final concentration of 0.5 mg mL^-1^ and incubated for 4 hours at 37 ºC. The purple-colored formazan salts formed were dissolved in DMSO, and the absorbance measured at 550 nm in a microplate reader (Synergy HT, Biotek, USA). A control without solvent, a solvent control (0.5% DMSO), and a positive control (20% DMSO) were included in every experiment.

**Anti-inflammatory activity**

Anti-inflammatory activity of **1** was tested against the murine RAW264.7 macrophage cell line. Cells were seeded in 500 µl of medium (complete DMEM as described above) in 24-well plates at a density of 4 x 10^5^ cells mL^-1^ and incubated for 24 hours to allow cell adhesion. Cells were challenged with lipopolysaccharides (LPS from *Escherichia coli* 0111:B4, Sigma) to induce inflammation, and co-exposed to **1** at a final concentration of 6 µM for 24 hours. A solvent control (DMSO, 0.3%) and a LPS-only control were included. Culture supernatant was added to Griess reagent (1% sulfanilamide, 0.1% N-(1-Naphthyl)ethylenediamine dihydrochloride dissolved in 5% phosphoric acid) in a ratio of 1:1 in a 96-well plate and incubated for 10 min in the dark. Absorbance was measured at 540 nm in a microplate reader (Synergy HT, Biotek, USA). A standard curve with sodium nitrite (Sigma) was performed with the Griess reagent in the same conditions in the range of 0.1 to 31 µM to determine the nitrite concentrations in the nitric oxide assay.

**Anti-obesity activity**

The anti-obesity activity of **1** was analyzed with the zebrafish Nile red assay as described in [1] with some modifications. Zebrafish adults and larvae were maintained under standard conditions at 28˚C as defined in the zebrafish book available at ZFIN database (https://zfin.org/). In brief, zebrafish embryos were raised from 1 DPF (days post fertilization) on in egg water (60 µg ml^-1^ marine sea salt dissolved in distilled H_2_O) with 200 µM PTU (1-phenyl-2-thiourea) to inhibit pigmentation. From 3 DPF to 5 DPF, zebrafish larvae were exposed to **1** at a final concentration of 2 µM with daily renewal of water and compound in a 24-well plate with a density of 15 larvae/well. A solvent control (0.1% DMSO) and positive control (REV, resveratrol, final concentration 50 µM) were included in the assay. Lipids were stained with Nile red overnight at the final concentration of 10 ng ml^-1^. For imaging, the larvae were anaesthetized with tricaine (MS-222, 0.03%) for 5 minutes and fluorescence analyzed with a fluorescence microscope (Leica DM6000B, Wetzlar, Germany). Fluorescence was quantified in individual zebrafish larvae by ImageJ (<http://rsb.info.nih.gov/ij/index.html)>.

**HDAC and 20S proteasome inhibition assays**

Compound **1** was evaluated for Histone Deacetylase (HDAC-Glo^TM^ I/II Assay and Screening System, Promega) and 20S proteasome (Proteasome-Glo^TM^ 3-Substrate System, Promega) activity inhibitions (chymotrypsin-, trypsin- and caspase-like) at a concentration of 10 μg ml^-1^ (11 μM), following the manufactures protocols. The inhibitors epoxomicin (0.5 μM) and trichostatin (50 and 5 nM) were used as positive controls for the 20S proteasome and HDAC assays, respectively. DMSO (1%, v/v) was used as a negative control. Luminescence was recorded in a microplate reader (Synergy HT, Biotek, USA) and the inhibition of the enzymes was determined as percentage taking into consideration 100% of the maximum signal.

**Anti-Quorum Sensing activity test**

The reporter strain *Chromobacterium violaceum* CECT 494 was used to determine the quorum sensing inhibition (QSI) activity of **1**, following the protocol developed by [2] with slight modifications. Briefly, *C. violaceum* CECT 494 was inoculated in 50 mL of Luria-Bertani (LB) broth (Difco) at an OD 600 nm of ~ 0.1 and grown overnight, at 26 ºC, in order to reach the exponential phase. The overnight culture was added to molten LB agar (5 ml; 0.3% w/v) and immediately poured over the surface of pre-warmed LB agar plates (Difco). Sterile paper-disks (Oxoid, Ldt., Wade Road, UK) were placed over the agar plates and impregnated with 15 µl of **1**, at a concentration of 10 µg mL^-1^ (11 μM) (1%, v/v), and the plates were incubated overnight at 26 ºC. Quorum-sensing inhibition was evaluated by the inhibition of violacein pigment production, represented by a colorless/opaque halo with viable bacterial growth. Growth inhibition representing antibacterial activity was indicated by a transparent halo around the discs representing no bacterial growth. An antibiotic cocktail (penicillin 5000 units mL^−1^, streptomycin 5 mg mL^−1^ and neomycin 10 mg mL^−1^, Sigma-Aldrich) was used as a positive control whereas DMSO (1%, v/v) served as a negative control.

**Screening for antibacterial activity**

Antibacterial activity of **1** was tested against five references strains, *Staphylococcus aureus* ATCC 25923, *Bacillus subtilis* ATCC 6633, *Enterococcus faecalis* ATCC 29212, *Escherichia coli* ATCC 25922 and *Pseudomonas aeruginosa* ATCC 27853. The antimicrobial activity was screened against all bacterial strains through the agar disc-diffusion method. Briefly, sterile blank paper discs (Oxoid Ldt., Wade Road, UK) were impregnated with 15 μL of the stock solution of **1**, at a concentration of 10 µg mL^-1^ (11 μM) (1%, v/v), and placed on Müller-Hinton (MH) plates previously inoculated with a bacterial inoculum equal to a 0.5 McFarland turbidity. The plates were then incubated at 37°C for 24 h and then the diameter of the zones of inhibition was measured in millimeters.

**Screening for allelopathic activity**

Compound **1** was tested at 30 μg mL^−1^ (33 μM) (from a stock solution, 1%, v/v) in anti-cyanobacterial and anti-algal bioassays that were carried out as previously reported [3]. Shortly, fresh culture stocks of the cyanobacteria; *Cyanobium* sp. LEGE 09398 and *Phormidium mougeotii* LEGE 07229 and the green microalgae; *Parachlorella* sp. LEGE Z-001 and *Ankistrodesmus falcatus* Z-006 were diluted in fresh culture medium (200 μL) to an OD 750 nm of ~ 0.1 (measured with the microplate lid on). Subsequently, the cultures were added (198 μL) to the microplate wells containing 2 μL of the stock solution of **1**. Optical densities (OD 750 nm) were measured in a microplate reader (Synergy HT, Biotek, USA) immediately after inoculation and following 7 days of exposure. An antibiotic cocktail (penicillin 5000 units mL^−1^, streptomycin 5 mg mL^−1^ and neomycin 10 mg mL^−1^, Sigma Aldrich), was used as positive control for the cyanobacterial assays whereas potassium dichromate (4 μg mL^−1^) was used as a positive control for the green microalgae assays. DMSO (1%, v/v) was used as a negative control for all the target organisms.

**References**

**1** K. S. Jones, A. P. Alimov, H. L. Rilo, R. J. Jandacek, L. A. Woollett and W. T. Penberthy, *Nutr. Metab.*, , DOI:10.1186/1743-7075-5-23.

**2** R. J. C. Mclean, L. S. Pierson and C. Fuqua, *J. Microbiol. Methods*, 2004, **58**, 351 – 360.

**3** P. N. Leão, V. Ramos, Gonçalves Patrício B., V. Flávia, O. M. Lage, W. H. Gerwick and V. Vasconcelos, *Mar. Drugs*, 2013, **11**, 1316–1335.
